# Effectiveness of a health promotion intervention to address determinants of child neglect in a disadvantaged community in Sri Lanka

**DOI:** 10.1186/s41043-021-00267-6

**Published:** 2021-11-08

**Authors:** Nadeeka Rathnayake, Shreenika De Silva Weliange, G. N. Duminda Guruge

**Affiliations:** 1 Department of Health Promotion, Faculty of Applied Sciences, Rajarata University of Sri Lanka, Mihintale, Sri Lanka; 2 Department of Community Medicine, Faculty of Medicine, University of Colombo, Colombo, Sri Lanka

**Keywords:** Child neglect, Community-centered intervention, Disadvantaged, Health promotion, Low and middle-income country, Victims and perpetrators of neglect

## Abstract

**Background:**

Child neglect is a form of child maltreatment and it is a neglected area of research. As similar to other forms of maltreatment, neglect also results in negative health outcomes for children. Child neglect is concentrated in disadvantaged communities. The community-centered health promotion approach empowers communities to identify and address determinants of perceived health issues. This study aimed to implement a health promotion intervention to enable a disadvantaged community to address determinants of child neglect and evaluate the effectiveness of the intervention.

**Methods:**

A quasi-experimental study design was used. Two disadvantaged communities in Anuradhapura District, Sri Lanka with similar socio-demographic characteristics were purposively selected as the experimental and control study settings. The mothers who have at least one child in the age range 5–18 years were included in the sample. The sample included 42 mothers from the experimental group and 44 mothers from the control group. The elder children of mothers in the experimental group were selected to be the agents of change. A health promotion intervention was implemented only with the experimental setting. The steps of the intervention included; (1) identifying prevention of child neglect as a goal, (2) understanding the determinants, (3) analyzing determinants and identifying actions and (4) implementing and modifying the actions. The total study duration was 1 year, with the intervention taking an average of 6 months. Data were collected at both pre and post-intervention phases from mothers and children through interviewer-administered questionnaires.

**Results:**

Mothers of the experimental group improved their knowledge on child neglect significantly in comparison to the control group (*p* < 0.05). In the post-intervention phase, there were significant differences in attitudes related to child neglect between experimental and control groups (*p* < 0.05). Following the health promotion intervention, mothers of the experimental group had significantly improved their practices related to the safety, education, nutrition of children, relationships with the child and social support for child caring (*p* < 0.05) when compared with the control group.

**Conclusions:**

The health promotion intervention was effective in addressing the selected determinants of child neglect in a disadvantaged community. Children could act as active agents of change to deliver the interventions to their mothers (perpetrators).

## Background

Many child maltreatment cases in Sri Lanka are unreported [1]. Child victims are not properly identified and even identified ones are released without appropriate therapies. This may lead the victim to become a perpetrator in the future [1]. Child abuse cannot be restricted only to physically visible signs such as bruises, wounds. Physical abuse is usually visible but other types of child abuse such as neglect too have many long-term negative consequences [2]. Child neglect is an under-researched area in East Asia and the Pacific region. Estimates of the prevalence of child neglect range from 22 to 32% for both genders in the region [3].

Child neglect is an under-studied area in child abuse though it is the commonest and deadliest form of child maltreatment [4]. Child neglect is associated with an inability to provide age-appropriate necessary care for children [5] and includes the omission of fulfilling children’s needs by caregivers where other forms of abuse include the caregiver commissions. Repeated omissions are categorized as neglect. When defining neglect, both the actual and potential harm on the child should be considered [4].

Child neglect is defined as, “failure to provide for the development of the child in all spheres: health, education, emotional development, nutrition, shelter and safe living conditions, in the context of resources reasonably available to the family or caretakers and causes or has a high probability of causing harm to the child’s health or physical, mental, spiritual, moral or social development” [6]. The same definition is used in the Sri Lankan national guideline for the management of child abuse and neglect [7]. In a Sri Lankan study [8], the considered indices of child neglect are the abandonment of children, failure to meet the basic needs of children and underutilization of the free services provided by the state [8]. Basically, child neglect is identified as the failure to provide children with basic needs such as food, clothing, hygiene and supervision [2]. The adverse effects of abuse or neglect of children vary depending on the circumstances of abuses, characteristics of children and the child’s environment [9].

There are different types of neglect. Physical neglect is the most highly recognized form of neglect. If the child is suffering or at risk due to the inability of caregivers to provide needs is referred to as physical neglect [10]. If caregivers are uninterested in the child and if the child is left alone for long periods, the child is prone to emotional neglect. Chronic truancy and continuous un-involvement of the child in educational programs come under educational neglect [10]. Inadequate supervision of children by caregivers which causes physical or psychological harm to children is identified as supervisory neglect [4]. Failure of parents to provide timely health care and medical recommendations for children are the indicators of medical neglect. If parents fail to consent to medical treatments that prevent the child from adverse conditions is also a component of medical neglect [10].

The factors that increase the susceptibility of children for neglect are called risk factors of child neglect [11]. For child neglect or abuse, a number of factors contribute and those are interconnected [10, 11]. Thus, the understanding of those factors is necessary to address the issue [11]. Many studies have identified poverty as the biggest causal factor of neglect [10]. Inadequate housing also causes neglect. Poor housing is always associated with poverty and other determinants of neglect [12]. Children in households with poor living conditions are at the risk of being neglected [13].

Lack of parenting skills is another cause for child neglect and especially teen parents neglect their children as they are unaware of how to raise their kids [2]. Parental substance abuse or dependencies are highly connected with child neglect independent of the confounding factors [14]. If children live in families experiencing violence and in families with many problems, they are neglected [12]. Caregivers with mental health problems fail to understand the importance of providing love, affection and other needs for children which causes child neglect [4]. Further, the parents who have been neglected as children in their past may pass their childhood experiences on to their children [2].

Social and environmental risk factors of child abuse and neglect include poor accessibility of child care and social services, lack of social support, discrimination, stressful life events and violent neighborhoods [15]. In a Sri Lankan study [8], poverty and ignorance have been identified as the main risk factors of neglect. The other factors that have been identified in the study are, cultural values, shortcomings in service delivery, female employment in both local and foreign environments and inadequate community services for child caring [8]. A large health impact is associated with child maltreatment. This impact may be either immediate, direct or more often be long-term, affecting the emotional development and overall health of children [16].

Child maltreatment is concentrated in disadvantaged environments [17]. Child neglect is mostly-reported in neighborhoods where stressful living conditions continue over time [18]. Child maltreatment rates are associated with the socio-economic features of neighborhoods. Neighborhoods sometimes support or act as barriers for children in families vulnerable to neglect [17]. The features of distressed neighborhoods that contribute to child neglect are, poor social support, high rates of truancy, juvenile arrests and teenage pregnancies [18, 19]. Impoverished communities lack positive formal or informal support for child caring [20]. Further, the children who live in unsafe neighborhoods are at a higher risk of neglect than children in safer neighborhoods [19].

There is a gap in the existing literature about the association between neighborhoods and child maltreatment [17]. Few examples of studies on child neglect in disadvantaged settings are; the stigma management strategies of a homeless, neglected group of children in the San Francisco bay area have been identified in a 4-year-long ethnographic study. In this study, strategies used by neglected children to fit into society have been identified [21]. Many researchers have reported that orphaned children in Sub-Saharan Africa are more prone to child neglect. Studies done in different countries of Sub-Saharan Africa have reported the presence of material and educational neglect [22]. There is a lack of awareness programs or interventions to intervene with child neglect in disadvantaged communities [22].

The selected community for the present study is a disadvantaged one. The ethnicity of a majority is Thelingu, which is an ethnic minority in Sri Lanka. The main occupation of mothers of this community is fortune-telling on the streets. Children accompany mothers on their jobs. Thus, most of the children are not schooling regularly. The children in the study setting in a way can be identified as street children. There are two categories of street children; children who live on the streets full time and children who work on the streets and return home at night. These children may do begging alone or with their caregivers [13, 23]. The children in this community belong to the second category as they stay with their mothers on the streets. This indicates that this community is deprived and different from other rural communities in Sri Lanka. This community can be identified as a transitory community group.

Caring for neglected children has a long history and different programs have been conducted by the governments, private and non-profit sectors. Some examples are a range of food programs including school breakfast programs [24]. In addressing and managing incidents of neglect, home visiting and establishing family support centers have been identified as effective methods [10, 11]. Parenting classes and caregiver support groups are great to learn parenting skills especially for teen parents [2]. Extremely poor families should receive support to make sure that their children are not neglected [12]. Child welfare systems extend services to neglected children but it is doubtful whether these programs reach those in need [24]. Even though some community-based programs have been done, there is a gap in research about experimental studies to address child neglect. The efforts of empowering communities to address child neglect by themselves are limited. Thus, the present study is about enabling a disadvantaged community to address determinants of child neglect.

Child neglect needs long-term interventions than ‘quick-fix’ solutions [10]. The interventions that target risk factors of child abuse and neglect need to be developed and tested. In addition, more concern should be paid to low-cost interventions directed at communities [25]. In the community-centered health promotion approach, community members take control of the factors that influence their lives and they improve their health and well-being [26]. The unique feature of the health promotion approach is being a community-owned, continuing process of improving wellbeing which is not only an activity or an event [26]. In the health promotion approach, ‘top-down’ approaches are replaced with community-centered ‘bottom-up’ ones [27]. Promoting the health of the communities goes beyond the health sector responses, making it a collective responsibility [28].

There is a lack of published studies regarding enabling disadvantaged community members in addressing determinants of child neglect. This study aimed to implement a health promotion intervention to enable a disadvantaged community to address determinants of child neglect and evaluate the effectiveness of the implemented intervention. In the present study, children who are the victims of neglect acted as agents of change. This study is a part of a multi-component study that aimed to address selected determinants of neglect of children aged 5–18 years in the Ihalaulpathwewa community in Anuradhapura district, Sri Lanka.

## Methods

### Study design

The quasi-experimental study design was used. The study was carried out in 3 phases; Phase I: Pre-intervention, Phase II: Intervention and Phase III: Post-intervention. In phase I, baseline data were collected from both experimental and control study settings. In phase II, the intervention was carried out only with the experimental study setting. In phase III, post-data were collected from both experimental and control study settings.

### Study settings

The Ihalaulpathwewa community in Mihintale Medical Officer of Health (MOH) area in Anuradhapura district was the experimental study setting while the Bendiwewa-Kudagama community in Thambuththegama MOH area in Anuradhapura district was the control study setting. The study settings were similar in socio-demographic characteristics. In both experimental and control study settings, the ethnicity of a majority is Thelingu which is an ethnic minority in Sri Lanka. People in both settings have access to very limited resources and their houses look like temporary huts. People have no access to drinking water supply and proper sanitary facilities within the villages. The main source of income of males is fishing and of females is fortune-telling on the streets. Both experimental and control settings lack health care centers and schools within the villages where they want to go to the nearest town to receive those services.

### Study population

All mothers who have at least one child in the age range of 5–18 years were included in the study population.

Cognitive, language and behavioral functioning of children aged 3–10 years with a history of neglect have been low compared to children without a history of neglect [29]. Further, it has been found hard for social care and other professionals to deal with the neglect of adolescents [30]. In Sri Lanka, only a little is known about the abuse and neglect that late adolescents experience and its long-term ill effects [31]. Thus, in the present study, the 5–18 age range was considered. Exclusion criteria were mothers who have children below 5 years and mothers who have children already married or living away from home. Mothers who have children below 5 years were not considered, as those children are very young to act as agents of change and as their developmental needs are different. The elder children (in the age range 5–18 years) of mothers in the experimental group were selected to be the agents of changing the process.

### Sampling method and sample size

Two study settings were selected purposively to carry out this study with disadvantaged communities. Eligible mothers (who have children in the age range 5–18 years) were identified from the details of *Grama Niladhari* (a government officer) and informal heads of villages. The first author reached out to the households of those mothers in person for two days to provide them with the information about the study and to obtain their consent. All eligible mothers in both experimental and control settings consented to participate. Then, the sample included 42 mothers from the experimental group and 44 mothers from the control group. The sample in settings varied because all who fulfilled eligibility criteria and provided the consent to participate were included in the sample as these are small communities and as this was a quasi-experimental study. The elder children of mothers in the experimental setting were recruited as agents of change with their consent if they were above 16 years. A parent’s consent was needed if the children were below 16 years. A total of 42 agents of change were recruited from the experimental group. In the experimental setting, all 42 mothers and 42 agents of change were followed up till the end of the study while one mother out of 44 mothers in the control setting was lost to follow up.

### Development of data collection materials

The data collection materials were developed based on expert guidance and literature review. Two interviewer-administered questionnaires were developed, one for mothers and the other for children. The questionnaires were reviewed and modified in two rounds with some experts in the field of child maltreatment prevention in Sri Lanka. Those questionnaires were pretested and discussed with a group of mothers and children who had similar characteristics to the study participants to get an understanding of the areas to be assessed under knowledge, attitudes and practices as well as to check the wording of the questions. Pre-testing helped to change the wording of the questionnaires as easily understood by mothers and children. Moreover, it helped to identify some specific attitudes and practices in these communities to be tested. With these outcomes of the pre-testing, some modifications were made in the questionnaires discussing with the experts.

### Data collection materials

*Interviewer administered questionnaire for mothers* included socio-demographic data in part I, questions related to knowledge of mothers about child neglect were included in part II, seven common attitudes were tested in part III and questions related to practices of mothers that contribute to child neglect were included in part IV.

Knowledge of mothers about child neglect was assessed in the following areas; whether they know who is a child (the age range), awareness of the needs of children, risk factors of neglect, warning signs of neglect and harmful effects of child neglect. Under harmful effects, separate questions were asked relevant to harm on child’s physical health, mental health, social health and education.

The tested attitudes were as follows; receiving basic needs is a right of a child, neglect is a huge problem for children, child neglect is a huge social issue, keeping children unsafe at home as parents leave for occupations is a problem, being in unhygienic conditions is a problem for children, child neglect can be avoided and parents should listen to children. Mothers had to express their level of agreement with these attitudes. The above attitudes, especially the ones related to hygiene and abandonment of children at homes can be identified as specific to selected study settings. Those were identified in a discussion with participants who were involved in pretesting of the questionnaires.

Mothers’ practices related to the safety of children were assessed using questions relevant to, leaving the child in unsafe conditions, paying adequate attention to illnesses of the child and discussing with the child in a friendly manner about how to deal with strangers and how to be careful when being alone at home. Practices related to the education of children were assessed using questions relevant to, providing a suitable home environment for children to learn, checking school homework, discussing schooling and education with the child and motivating the child to go to school daily.

Practices related to the hygiene of children were assessed using questions relevant to, searching and taking care of the physical cleanliness of the child and discussing the physical cleanliness with the child. Practices related to the nutrition of children were assessed using questions relevant to, checking whether the child has eaten enough, considering the nutritious value of the meal that the child takes and discussing the harm of junk food consumption with the child. Practices related to the relationship between the mother and the child were assessed using questions relevant to, spending a satisfying time with the child, spending the time effectively with the child, appreciating the good work of the child and discussing with the child and finding solutions for problems of the child. Social support received for child caring was assessed using questions relevant to, whether neighboring mothers care about safety, education and physical cleanliness of your child, whether neighboring mothers share meals with your child, pay attention to illnesses of your child, appreciate the good work of your child and discuss friendly with you about fulfilling needs of your child.

*Interviewer administered questionnaire for children* included two parts. Part I included questions related to unity and caring among children whereas part II included questions related to practices of mothers that contribute to child neglect.

Unity and caring among children are important in addressing child neglect as a group and sustaining the intervention in the community. Working as a group of change agents helps improving unity and caring among children. Thus, the unity and caring among children were assessed in the areas of; sharing meals with friends, helping friends for learning, assisting friends in troubles, concerning about hygiene of friends, caring at illnesses of friends, whether children feel safe and happy with friends and ability to communicate comfortably with friends.

In part II, the same questions which were asked from mothers about practices were asked from children as well to find out whether their mothers engage in those practices.

### Development and the implementation of the intervention

**Fig. 1:**
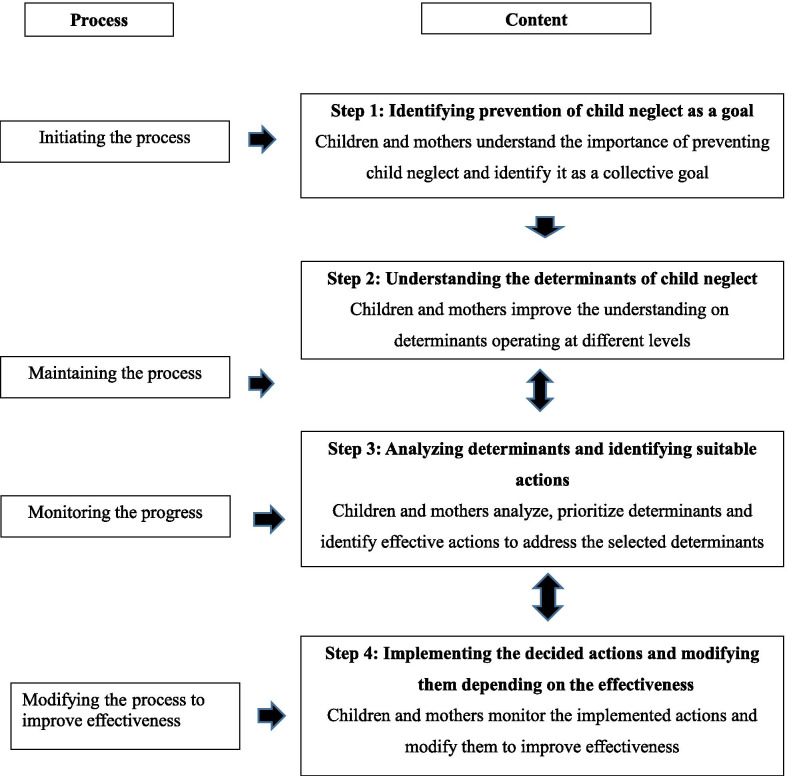
The conceptual framework for the intervention [26]

The conceptual framework for the intervention (Fig. 1) was adopted from the community-centered health promotion intervention model [26] which had been subsequently used in several studies [32, 33]. The model consists of two components as process and content. The process gives the flow of the intervention while content gives the subject matter.

### Step 1: Identifying prevention of child neglect as a goal

A children’s society was initiated with the agents of change and a simple tool namely ‘happiness calendar’ [32] was introduced. Children were guided to mark their everyday moods (for example, whether they were happy, sad or angry) using the calendar. After about a week, the first author visited the community, assessed the progress and discussed the reasons for the unhappiness. It was understood that not meeting their basic needs is one of the major reasons for the unhappiness of children. Next, the first author discussed the topic of ‘child neglect’ with children. Two discussions were facilitated with children on what is neglect, warning signs and harm of neglect and then, it became a collective goal to be addressed. Once children understood the importance and possibility of addressing child neglect within their community, they easily switched to be agents of change from the role of victims. The first author guided children continuously on how to reach mothers, conduct discussions with mothers and monitor the ongoing changes as agents of change at children’s society meetings. Then, another two discussions were facilitated with respective mothers of the experimental group about child neglect. These discussions were mainly moderated by children using simple techniques such as using pictures and small group activities. For example, children developed picture cards to discuss their basic and emotional needs and also warning signs of neglect. The first author provided the inputs, guided the group by providing clarifications for both children and mothers when necessary. After those two discussions, mothers also understood the importance of preventing child neglect. Each discussion was conducted for an hour and at convenient times for children and mothers mainly at the village church and sometimes under a shade of a tree.

### Step 2: Understanding the determinants of child neglect

In this step, the first author conducted a 1-h group discussion with the children about the determinants that contribute to child neglect. To make the process easier, children were divided into five sub-groups considering their age and gender. Each group was asked to discuss and mention the determinants of neglect that they could identify. Thereafter, the first author facilitated more information based on the determinants reported in the existing literature. Children were able to identify both visible and unseen determinants of neglect. Children above 10 years of age conducted another 1-h group discussion with mothers regarding the importance of identifying the determinants. Children used ‘story cards’ on which the stories of child neglect were written by themselves to facilitate that discussion with mothers with the guidance of the first author. As a result, mothers also could identify determinants of child neglect.

### Step 3: Analyzing determinants and identifying suitable actions

The study sample including both children and mothers collectively analyzed and prioritized the identified determinants. Three determinants were prioritized to be addressed in a discussion with the first author. These include poor practices of mothers inherited by their culture, poor mother–child relationships and poor social support. With the technical inputs of the first author, children could design 14 interventions to address the prioritized determinants (Table 1).

**Table 1:** Summary of the interventions implemented to address prioritized determinants

Intervention	Description	Prioritized Determinants 1. Poor practices of mothers 2. Poor mother–child relationships 3. Poor social support		
		1	2	3
1. Informal discussions	Children started discussing with mothers about their poor practices, poor relationships with children and poor social support that tend to child neglect in informal settings such as in common bathing place and on the way to fortune-telling. Those discussions were marked on a village map.	×	×	×
2. Peer group discussions by mothers	Children identified that mothers also can carry on this process of informal discussions with their peer mothers. Then, children identified the enthusiastic mothers. Children communicated about the process to enthusiastic mothers and they too joined in the process. Informal discussions by mothers too were marked on the map.	×	×	×
3. Poster campaign	Children prepared some posters that depict healthy children and supportive communities. These posters had only pictures and no words. They pasted these posters in common places in the village where everyone can see those.	×	×	×
4. Discussions with mothers by children	Children arranged discussions with mothers’ groups. In these discussions, children talked about the needs of children using ‘needs and wants’ cards. Children discussed signs and harm of child neglect using pictures with mothers. In addition, children discussed all three prioritized determinants of child neglect and changes among mothers, families and community.	×	×	×
5. Support groups	Mothers’ groups were developed as support groups. Mothers started helping each other in their support group, especially for child caring.			×
6. “Ammail-Mamai” (mum and me) and “Ammai- Daruwai” (mum and child) tools	Using the “Ammai-Mamai” tool, children gave marks for the practices of mothers and using the “Ammai-Daruwai” tool, mothers gave marks for their practices.	×	×	
7. “Api dan sameepai” (We are close) tool	Using the tool, mothers and children identified and marked their relationships once a fortnight.		×	
8. “Apita-Api” (We for We) tool	Mothers measured the extent of social support that they gain to raise their children by using this tool.			×
9. Public displaying of measurements by children	Children’s marks for practices of mothers and relationships with mothers were displayed publicly without names.	×	×	
10. Child- friendly home	Children started observing and giving marks out of ten for each other’s home. They observed whether the home has a suitable environment for learning, whether the home ensures the child’s safety etc. They observed the surroundings of the home too. They pasted the mark written on a white paper on a wall of the home.	×	×	
11. Environmental cleanliness and home gardening	Mothers and children arranged campaigns to clean common places in the village and started doing home gardening.		×	×
12. Food sharing	Children started sharing food with friends and they offered food to their friends if they found nothing to eat at home. This practice was communicated to support groups of the mothers and they too started it.	×	×	×
13. Collective learning	Elder children in the village started two classes for smaller children free of charge within the village premises. One class was in the church on Sundays and another one was in the community hall on Wednesday and Friday evenings. Mothers brought nicely dressed children with books and pencils to these classes.	×	×	×
14. Learning through games	Mothers developed some games using numbers and letters that the children can play and learn**.** Sometimes mothers too played the games with children. The games were played mainly in children’s society meetings.	×	×	×

### Step 4: Implementing the decided actions and modifying them depending on the effectiveness

Children started implementing the interventions listed in Table 1 with the continuous inputs by the first author. They were able to guide and encourage their mothers to implement possible actions. Ten more discussions led by the children were conducted with mothers during which they identified and measured the changes in determinants as a result of the interventions. The progress of the interventions was reported in an anonymous way discussing with children and mothers. All the changes encountered and necessary modifications to be made in interventions were discussed by children with the first author on weekly basis at their children’s society meetings.

At the end of the intervention, the activities implemented and changes that happened in the experimental setting were shared with the control group in a forum led by the first author. It supported the control group as well as initiating some activities to address child neglect in their community.

### Data collection

Data collection was done by the first author at home visits from both experimental and control study settings using interviewer-administered questionnaires for mothers and children. The same questionnaires that were used in phase I was repeated in phase III.

### Data analysis

Data were analyzed using SPSS version 21. To analyze the level of knowledge of mothers about child neglect, 1 mark was given for each correct answer and 0 marks was given for a wrong answer or if they didn’t know the answer. Practices were scored from 0 to 3 marks (0—‘Never’, 1—‘Rarely’, 2—‘Sometimes’ and 3—‘Most often’) for each item. Thereafter, total knowledge and practice scores were computed by adding the item scores and then converted to percentage scores. This approach was used for both the experimental and control groups before and after the intervention. Independent samples *t*-test was used to assess the difference in knowledge and practice scores among the experimental and the control groups both before and after the intervention. Thereafter, paired *t*-test was used in the two groups to see if a significant change occurred before and after the intervention. Further, for each group the difference in the knowledge and practice scores were calculated, independent samples t-test was applied to this ‘difference in the scores’. The attitudes of mothers were analyzed by recording the variables ‘strongly agree and agree’ as favorable attitudes and ‘neutral, disagree and strongly disagree’ as unfavorable attitudes. Differences between the attitudes in the experimental and control groups were assessed using chi-square statistics.

## Results

### Socio-demographic data of study participants

Socio-demographic information of the experimental and control groups is given in Table 2. Out of 42 mothers in the experimental group, 40.5% were in the age group of 21–25 years and out of 44 mothers in the control group, 36.4% were in the 21–25 age group. 83.3% of mothers in the experimental group and 77.3% of mothers in the control group had never been to school. A majority of mothers in both experimental (64.3%) and control (70.5%) groups were fortune-tellers and it was their main source of livelihood. The ethnicity of a majority of participants in both groups is ‘Thelingu’. 83.3% and 75% of mothers in experimental and control groups respectively were in households with a monthly income below 5000LKR (25.07USD). There were no significant differences in socio-demographic data of the experimental and control groups.

**Table 2:** Socio-demographic data of study participants in the experimental and control groups

	Experimental group		Control group		Sig
	Number	%	Number	%	
*Socio-demographic data of mothers*					
Age					
< 20	6	14.3	6	13.6	*p* = 0.553 × 2 = 3.029 df = 4
21–25	17	40.5	16	36.4	
26–30	11	26.2	12	27.3	
31–35	6	14.3	10	22.7	
> 36	2	4.8	0	0	
Education					
Not schooled	35	83.3	34	77.3	*p* = 0.778 × 2 = 0.502 df = 2
Grade 1–5	5	11.9	7	15.9	
Grade 6–11	2	4.8	3	6.8	
Occupation					
Fortune telling	27	64.3	31	70.5	*p* = 0.779 × 2 = 1.765 df = 4
Selling incense sticks	9	21.4	8	18.2	
Selling books in buses	3	7.1	4	9.1	
Other	2	4.8	1	2.3	
No job	1	2.4	0	0	
Ethnicity					
Thelingu	37	88.1	38	86.4	*p* = 0.810 × 2 = 0.058 df = 1
Sinhala	5	11.9	6	13.6	
Other	0	0	0	0	
Religion					
Christianity	37	88.1	40	90.9	*p* = 0.670 × 2 = 0.182 df = 1
Buddhism	5	11.9	4	9.1	
Other	0	0	0	0	
Monthly income of household					
< 5000	35	83.3	33	75	*p* = 0.652 × 2 = 1.632 df = 3
5000–10,000	6	14.3	8	18.2	
10,000–20,000	1	2.4	2	4.5	
20,000–30,000	0	0	1	2.3	
> 30,000	0	0	0	0	
No. of children					
< 2	20	47.6	19	43.2	*p* = 0.171 × 2 = 0.918 df = 2
3–4	14	33.3	16	36.4	
> 5	8	19	9	20.5	
Marital status					
Married	24	57.1	28	63.6	*p* = 0.546 × 2 = 4.025 df = 5
Unmarried	2	4.8	0	0	
Divorced	1	2.4	2	4.5	
Widowed	1	2.4	3	6.8	
Separated	12	28.6	9	20.5	
Remarried	2	4.8	2	4.5	

Before the health promotion intervention, the knowledge scores were almost similar in the experimental and the control groups (*p* > 0.05). Mothers of the experimental group have significantly improved (*p* < 0.05) their knowledge level regarding child neglect after the health promotion intervention according to percentage scores (Table 3). The highest mean is reported regarding knowledge on ‘needs of children’ in both pre and post-assessments. There are no significant improvements of knowledge of mothers in the control group except the knowledge on harm on social health (*p* = 0.024) and education (*p* = 0.032). According to reported p values, the improvement of knowledge of mothers in the experimental group is significant compared to the control group regarding all assessed aspects except knowledge on warning signs of neglect (*p* = 0.127 > 0.05).

**Table 3:** Knowledge of mothers about child neglect

Knowledge on child neglect	Experimental (*n* = 42)		Control (*n* = 43)		*p* value for the ‘difference in the scores’ between experimental and control groups**
	Pre Mean (SD)	Post Mean (SD)	Pre Mean (SD)	Post Mean (SD)	
Needs of children	25.39 (10.49)	51.05 (16.47)	25.58 (9.85)	28.42 (11.95)	p < 0.001
	p < 0.001*		p = 0.070*		
Risk factors of neglect	14.76 (11.52)	40.95 (15.74)	16.74 (10.40)	19.53 (10.90)	p < 0.001
	p < 0.001*		p = 0.063*		
Warning signs of neglect	14.88 (16.61)	23.21 (20.20)	12.79 (13.77)	15.11 (18.20)	p = 0.127
	p = 0.025*		p = 0.160*		
Harm on physical health	7.73 (16.08)	27.97 (21.52)	8.13 (21.63)	9.88 (16.49)	p < 0.001
	p < 0.001*		p = 0.583*		
Harm on mental health	7.14 (13.11)	23.33 (18.69)	7.90 (20.53)	11.16 (19.17)	p < 0.001
	p < 0.001*		p = 0.146*		
Harm on social health	8.33 (21.85)	30.95 (36.54)	5.81 (19.54)	11.62 (26.36)	p = 0.006
	p < 0.001*		p = 0.024*		
Harm on education	12.69 (22.02)	35.71 (27.93)	3.10 (12.20)	7.75 (17.574	p < 0.001
	p < 0.001*		p = 0.032*		

*Paired *t*-test; **Independent sample *t*-test (df = 83), equal variances not assumed

There are significant differences (*p* < 0.05) between all attitudes of mothers in the experimental and the control groups in the post-intervention phase (Table 4). The percentage of mothers who had favorable attitudes is higher in the experimental group compared to the control group.

**Table 4:** Attitudes regarding child neglect in the post-intervention phase

Attitudes of mothers						
		Experimental group		Control group		Sig
		Number	%	Number	%	
1. I think that receiving food, housing, protection, health care and education is a right of a child	Favorable	18	42.9	7	16.3	*p* = 0.007 × 2 = 7.229 df = 1
	Unfavorable	24	57.1	36	83.7	
2. I believe that child neglect is a huge problem for children	Favorable	10	23.8	3	7	*p* = 0.031 × 2 = 4.647 df = 1
	Unfavorable	32	76.2	40	93	
3. I think that child neglect is a huge social issue	Favorable	15	35.7	7	16.3	*p* = 0.041 × 2 = 4.184 df = 1
	Unfavorable	27	64.3	36	83.7	
4. I think that keeping children unsafe at home as parents leave for occupations is a problem	Favorable	11	26.2	2	4.7	*p* = 0.006 × 2 = 7.609 df = 1
	Unfavorable	31	73.8	41	95.3	
5. I believe that being in unhygienic conditions is a problem for children	Favorable	13	31.0	3	7	*p* = 0.005 × 2 = 7.993 df = 1
	Unfavorable	29	69.0	40	93	
6. I think that child neglect can be avoided	Favorable	14	33.3	6	14	*p* = 0.035 × 2 = 4.435 df = 1
	Unfavorable	28	66.7	37	86	
7. I believe that parents should listen to children	Favorable	7	16.7	1	2.3	*p* = 0.024 × 2 = 5.125 df = 1
	Unfavorable	35	83.3	42	97.7	

Before the health promotion intervention, the practice scores were almost similar in the experimental and the control groups (*p* > 0.05). According to both mothers and children, mothers of the experimental group have significantly improved their practices (*p* < 0.01) after the health promotion intervention except the practices related to ‘hygiene of children’ (Table 5). The mothers in the control group have not significantly improved their practices (*p* > 0.05). The improvement of practices of mothers in the experimental group is significant (*p* < 0.05) compared to the control group except for practices related to ‘hygiene of children’.

**Table 5:** Practices of mothers related to child neglect (according to mothers and children)

Practices	Experimental (*n* = 42)		Control (*n* = 43)		*p* value for the ‘difference in the scores’ between experimental and control groups**
	Pre Mean (SD)	Post Mean (SD)	Pre Mean (SD)	Post Mean (SD)	
*Practices related to child neglect (according to mothers)*					
Safety of children	13.17 (20.84)	28.73 (20.05)	11.47 (18.47)	13.95 (17.68)	p < 0.001
	p < 0.001*		p = 0.073*		
Education of children	5.15 (11.33)	16.46 (21.58)	12.01 (19.44)	12.79 (12.38)	p = 0.005
	p = 0.001*		p = 0.708*		
Hygiene of children	8.73 (19.560)	13.88 (19.44)	14.34 (22.29)	15.89 (20.87)	p = 0.246
	p = 0.062*		p = 0.323*		
Nutrition of children	8.73 (14.21)	33.86 (25.84)	11.88 (18.84)	13.69 (17.45)	p < 0.001
	p < 0.001*		p = 0.128*		
Relationships with the child	7.93 (13.76)	28.17 (19.38)	10.85 (15.27)	12.59 (11.69)	p < 0.001
	p < 0.001*		p = 0.254*		
Social support for child caring	8.39 (16.985)	26.53 (16.86)	7.19 (13.20)	8.41 (9.51)	p < 0.001
	p < 0.001*		p = 0.398*		
*Practices related to child neglect (according to children)*					
Safety of children	10.79 (18.62)	30.63 (21.340)	9.61 (17.59)	11.47 (15.349)	p < 0.001
	p < 0.001*		p = 0.462*		
Education of children	5.35 (12.72)	15.87 (20.31)	9.88 (19.60)	11.62 (16.77)	p = 0.030
	p = 0.001*		p = 0.523*		
Hygiene of children	6.34 (14.702)	10.31 (16.02)	9.30 (19.34)	10.46 (15.00)	p = 0.449
	p = 0.077*		p = 0.696*		
Nutrition of children	7.67 (16.55)	34.12 (24.71)	10.07 (18.75)	10.85 (13.38)	p < 0.001
	p < 0.001*		p = 0.778*		
Relationships with the child	5.55 (12.02)	28.96 (17.28)	9.30 (16.98)	10.27 (10.88)	p < 0.001
	p < 0.001*		p = 0.628*		
Social support for child caring	7.70 (16.49)	26.75 (16.15)	7.08 (14.56)	8.41 (9.457)	p < 0.001
	p < 0.001*		p = 0.477*		

*Paired t-test; **Independent sample *t*-test (df = 83), equal variances not assumed

Unity and caring among children who acted as agents of change in the experimental group have been significantly improved (*p* < 0.01) while there is no significant improvement (*p* > 0.05) in the control group (Table 6). The improvement of unity among children in the experimental group is significant compared to the control group (*p* < 0.01).

**Table 6:** Unity and caring among children who acted as agents of change

Unity among children	Experimental (*n* = 42)		Control (*n* = 43)		*p* value for the ‘difference in the scores’ between experimental and control groups**
	Pre Mean (SD)	Post Mean (SD)	Pre Mean (SD)	Post Mean (SD)	
Unity and caring	13.39 (10.10)	39.58 (17.22)	12.50 (11.88)	11.62 (12.01)	p < 0.001
	p < 0.001*		p = 0.719*		

*Paired *t*-test; **Independent sample *t*-test (df = 83), equal variances not assumed

## Discussion

Child neglect has received a least community and scientific attention though it also has worse long-term consequences similar to sexual or physical abuse [4, 34]. Child neglect is an under-researched area in child maltreatment [4]. The difficulty in detecting neglect can be a reason for the less attention paid to child neglect by researchers [5]. Evidence on effective strategies for the prevention of child maltreatment mostly comes from high-income countries [35]. Thus, it is a challenge to design interventions in low and middle-income countries to address child maltreatment. Although Sri Lanka is taking efforts to prevent all forms of child abuse, insufficient information is available on child neglect [13].

Child maltreatment is concentrated in disadvantaged environments [17] and it is a strength of the present study to conduct it in a disadvantaged setting. This study is unique because children who are the victims of neglect were the agents of changing the process. Moreover, both victims and perpetrators were involved. To implement effective interventions, the underlying factors of neglect should be well understood [10]. The specific risk factors that prevail within families for child neglect should be considered when designing prevention strategies [36]. Ministry of Child Development and Women’s Affairs of Sri Lanka has also taken many actions such as building safe houses for orphaned and neglected children whose needs are not fulfilled [37], but the efforts of enabling communities to address the root causes of neglect are poor. Further, there is a lack of published studies available on community-based interventions where communities are empowered to change determinants of child neglect. In the present study, identification of determinants of child neglect with the community before designing the interventions can be identified as a strength.

According to the results of the present study, mothers in the experimental group have significantly improved their knowledge on child neglect compared to the control group except the knowledge on warning signs of neglect. The highest mean was reported regarding knowledge on the needs of children. This study shows that simple health messages can be effectively delivered to mothers or caregivers who have received less formal education using pictorial cards in interactive group discussions. However, the groups need to be small for everyone to see the pictures and sessions should be short with a key take-home message [38]. Children were able to use such strategies as suitable for the educational level of mothers to provide them with the knowledge. As an example, children used some ‘picture cards’, on which they drew their ‘needs’ and ‘wants’ and asked their mothers to select the cards with the needs of the children. These activities generated the enthusiasm of mothers and were effective in delivering simple messages. In the post-intervention phase, mothers in the experimental group have significantly changed their attitudes related to child neglect in comparison to the control group.

The use of a control group to evaluate the effectiveness of the intervention and mobilizing children to be the agents of change are also the strengths of the study. Another strength in the study was to assess changes in practices of mothers from both mothers and children. According to the perspectives of both mothers and children, similar findings were observed related to practices of mothers that contribute to child neglect. Mothers in the experimental setting had significantly improved all assessed practices related to safety, education, nutrition, relationships with the child and social support for child caring proving the effectiveness of the implemented health promotion intervention. In the category of ‘social support for child caring’, the questions related to support extended by neighboring mothers for child caring were asked from mothers. Using these questions, the community level changes could be assessed beyond the individual or family levels.

However, there were no significant improvements in the practices of mothers related to the hygiene of children. Perhaps this could be due to a gap in the data collection instrument used because only a few questions about the practices of mothers related to the hygiene of children were asked. Those questions may be inadequate to assess all changes related to the hygiene of children. This is a common weakness in this kind of community-based interventions where all changes that encounter after the intervention cannot be assessed by using the same data collection instruments that were used in the pre-intervention phase.

The knowledge on the harm of neglect on social health and education has been significantly improved in the control group even without intervention. Perhaps, it may be due to the country-wide awareness programs on the importance of providing school education to all children and different programs conducted by the media on child maltreatment prevention. However, all other changes that happened in the control group were insignificant. The first author asked similar questions during pre and post-intervention phases from both experimental and control groups which also helped to reduce biases in data collection.

Positive impacts have been claimed with programs that enhance early family support to prevent child neglect. With the increase in family support, there has been a prominent reduction in neglectful behaviors as well as an increment in the caregiver–child relationship and family functioning [39]. In the present study even, social support and mother–child relationship were two of the addressed determinants and significant changes were found in practices of mothers that lead to child neglect.

Some other possible emerging interventions to reduce child neglect include, supporting parenting education, joining families with formal or informal support systems, coupling long-term interventions with professional helpers and providing health education to reduce the risk for child neglect [40]. A study [41] has shown that the families which have received home visits during pregnancy and infancy have fewer child maltreatment incidents reported involving the mother as the perpetrator and the child as the victim in comparison to other families without home visits [41]. Family group conferencing is a measure that improves the safety of the child and strengthens the family and community support networks. This has bought community members and professionals together to assist parents and children who are at risk of neglect [24]. The interventions implemented in the present study shared some features of the above programs such as visiting the homes of mothers, increasing social support and providing education to mothers on child neglect. However, there are wide differences in methods and effectiveness of community-based interventions to prevent child maltreatment [42] which make it difficult to do comparisons and identify the most effective interventions.

It is doubtful whether the child welfare systems effectively respond to the needs of poor neglect victims. Depending only on traditional caring practices such as foster care seems to be inadequate for the issue of child neglect [24]. New approaches should be developed and tested. The families where neglect presents need long-term solutions [10]. Discussions with civil society groups and communications with children and parents are to be facilitated when working on issues related to all forms of child maltreatment [35]. Community involvement should be made to assess environmental and home conditions that lead to child neglect [10]. In the present study, the community-centered health promotion approach was used and active community involvement was made to address the matter of neglect which is a strength of the study.

Despite all the strengths of this study, there are some limitations. In the present study, the self-reported improvements in knowledge, attitudes and practices by mothers and children are reported without an assessment of the real reduction in the incidence of child neglect which is a limitation. However, as there are significant improvements in the practices of mothers, it can be assumed that cases of child neglect would be reduced. Further, the structural determinants of child neglect such as poverty, poor housing and poor accessibility to services have not been addressed in the present study other than the modifiable determinants by the community; poor practices, social support and the mother–child relationship as per the aim of the study. There is a substantial overlap between child neglect and poverty. Studies have found that neglected children also experience harmful effects similar to poor children [24]. However, all children who live in poverty are not neglected. Research benefit greatly to develop interventions for poor children to overcome neglect [25], where this study also can be one such research. In this study, there is no fathers’ involvement to address child neglect which is also a limitation.

Researchers from different disciplines need to collaborate to identify root causes and consequences of child neglect and new directions should be identified [43]. Continuous and effective efforts should be taken along with all sectors of society including pediatricians, mental health professionals, counselors, community health workers, teachers, law enforcement agencies, religious leaders and communities to address the matter of child neglect [10, 44].

Although the National Child Protection Authority (NCPA) in Sri Lanka is taking efforts to address child abuse, data are based on reporting evidence but there may be many unreported cases. At the same time, some data are not generalizable due to small samples [13]. Based on the findings of the present study, the current interventions implemented by the NCPA and other field staff in Sri Lanka in addressing child neglect are to be modified with the active involvement of victims and perpetrators of neglect. Furthermore, children who are the victims of neglect are to be involved in the process of addressing child neglect after taking necessary actions to rehabilitate them if required and they should move away from the typical role of beneficiaries.

## Conclusions

Based on the findings of the study, it can be concluded that disadvantaged community members were enabled to address determinants of child neglect and the community-centered health promotion intervention was effective in guiding the community members. The children who are the victims of neglect could act as active agents of change and they delivered interventions effectively to their mothers. A majority of mothers who were involved with the study had not schooled and they received a very low monthly income however, they could address determinants of child neglect with proper guidance. The technical guidance to initiate and continue the intervention was relatively small, but it was systematic and culturally appropriate. Empowering victims is an advantage and can even guarantee the sustainability of the process in the community because victims have the perceived need to get the issue addressed.

The key recommendations that can be drawn from the study are as follows; it would be better if health promotion interventions could be coupled with initiatives to address structural determinants of neglect such as poverty, poor housing in future research and the findings from the study will be helpful for Sri Lankan policy-makers and administrators to develop effective measures to reduce child neglect. NCPA officers and other field officers who work on child neglect in Sri Lanka need to be trained on the community-centered health promotion approach and officers should be equipped with skills to enable victims and perpetrators of neglect to address the underlying determinants. As well as, it will be worth involving fathers and other family members in interventions that address child neglect in addition to the mothers. At the same time, a special focus is to be given to disadvantaged communities and settings where child neglect is highly prevailing or at a higher risk for neglect.

## Data Availability

The dataset used and analyzed during the current study is available from the corresponding author on reasonable request.

## Abbreviations

MOH: Medical Officer of Health
NCPA: National Child Protection Authority
